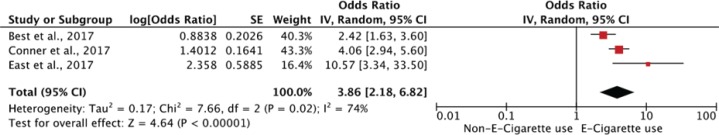# Corrigendum: Is adolescent e-cigarette use associated with smoking in the United Kingdom?: A systematic review with meta-analysis

**DOI:** 10.18332/tpc/114021

**Published:** 2019-11-06

**Authors:** Adewale Aladeokin, Catherine Haighton

**Affiliations:** 1 Northumbria University, Newcastle, United Kingdom

**Keywords:** adolescent, e-cigarette, United Kingdom, systematic review, smoking

**Corrigendum on:**

Is adolescent e-cigarette use associated with smoking in the United Kingdom?: A systematic review with meta-analysis By Adewale Aladeokin, Catherine Haighton Tobacco Prevention and Cessation, Volume 5, Issue April, Pages 1-13 Publish date: 22 April 2019 DOI: https://doi.org/10.18332/tpc/108553

An error in data entry occurred during the production of Figure 3 in the manuscript, as the authors accidentally entered the published adjusted ORs from three included studies as Log Odds in the generic inverse variance meta-analysis. Correcting the error shows that the OR remains significant: 3.86; p<0.00001 instead of 26.01[5.35, 126.44]. The conclusions were based on the meta-analysis of unadjusted ORs which was higher than this (OR: 5.55). However, as people are reportedly dying from severe lung - related illnesses tied to e-cigarettes and many countries are banning e-cigarettes it remains important to review the evidence that is currently available.

Figure 3: Meta-analysis based on adjusted odds ratios

The figure is the following:

**Figure:**
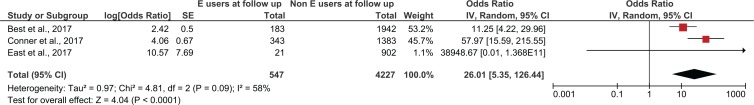


The correct figure should be:

**Figure:**